# Chemical Oscillation and Morphological Oscillation in Catalyst-Embedded Lyotropic Liquid Crystalline Gels

**DOI:** 10.3389/fchem.2020.583165

**Published:** 2020-10-23

**Authors:** Guanying Li, William Cortes, Qizheng Zhang, Ye Zhang

**Affiliations:** Bioinspired Soft Matter Unit, Okinawa Institute of Science and Technology Graduate University, Okinawa, Japan

**Keywords:** belousov-zhabotinsky reaction, lyotropic liquid crystalline gels, non-covalent catalyst-embedment, chemical oscillation, morphological oscillation

## Abstract

Liquid crystalline gels offer promising means in generating smart materials due to programmable mechanics and reversible shape changes in response to external stimuli. We demonstrate a simple and convenient method of constructing catalyst-embedded lyotropic liquid crystalline (LLC) gels and achieve chemomechanical oscillator by converting chemical waves in Belousov–Zhabotinsky (BZ) reaction. We observe the directed chemical oscillations on LLC sticks accompanied by small-scale oscillatory swellings–shrinkages that are synchronized with the chemical waves of an LLC stick. To amplify the mechanical oscillations, we further fabricate small LLC fibers and achieve macroscopically oscillatory bending–unbending transition of the LLC fiber driven by a BZ reaction.

## Introduction

Belousov–Zhabotinsky (BZ) reaction performed in low-density media such as in solution gives fascinating rhythmic color oscillations and spatiotemporal patterns. In a soft matter, typically in gels, chemical oscillation can be converted into mechanical motion by periodic gel–sol transition, swelling, and contraction. Since Yoshida et al. first demonstrated a copolymer gel as chemomechanical oscillator (Yoshida et al., 1996), approaches of self-assembly of copolymers (Yoshida and Ueki, 2014; Kim et al., 2017), cross-linking polymers (Zhang et al., 2013a, 2014), branched polyethylene glycols (PEGs) (Ueki et al., 2014), and the use of gelatin (Smith et al., 2013; Buskohl and Vaia, 2016) have been widely adapted to make self-oscillating BZ gels. We have also developed post-self-assembly cross-linking approach to construct mixed peptide–polymer oscillatory gels (Zhang et al., 2012a, 2013b). Autonomous gels that can self-oscillate driven by BZ reaction have demonstrated great potential in the design of smart materials, such as biomimetic actuators, mass transport surface, and oscillatory reactors (Yoshida, 2010; Yoshida and Ueki, 2014; Kim et al., 2017; Hou et al., 2019; Cheng and Perez-Mercader, 2020).

Liquid crystals self-organize at the molecular level while maintaining fluidity. Because of the anisotropic orientation, liquid crystals are sensitive to external fields. Assembly of liquid crystals to form liquid crystalline gels can further sensitize their response to stimuli, giving rise to macroscale mechanical responses, such as bending, twisting, and buckling (Ware et al., 2015; White and Broer, 2015). In this regard, liquid crystalline gels are the ideal soft material to construct BZ reaction driving mechanical oscillators (Bléger, 2018). To our surprise, only a few attempts of incorporating chemical oscillations into liquid crystal system have been carried out (Balasubramanian and Rodley, 1991; Shintate et al., 2018), and no example has ever been proposed for self-oscillating liquid crystals in BZ reaction.

We hypothesize that the oxidation–reduction cycle of metal catalyst in the BZ system will locally change the hydrophobic/hydrophilic environment, inducing novel orientations of mesogens in liquid crystalline gels and macroscopically self-oscillating by converting the continuous chemical waves. Herein for conceptual proof, we demonstrate a simple and convenient method of constructing catalyst-embedded lyotropic liquid crystalline (LLC) gels and realize macroscopic mechanical oscillations of bending–unbending transition driven by BZ reaction in LLC gels for the first time.

## Results and Discussion

### Design of Catalyst-Embedded LLC Gels

**Figure 1:**
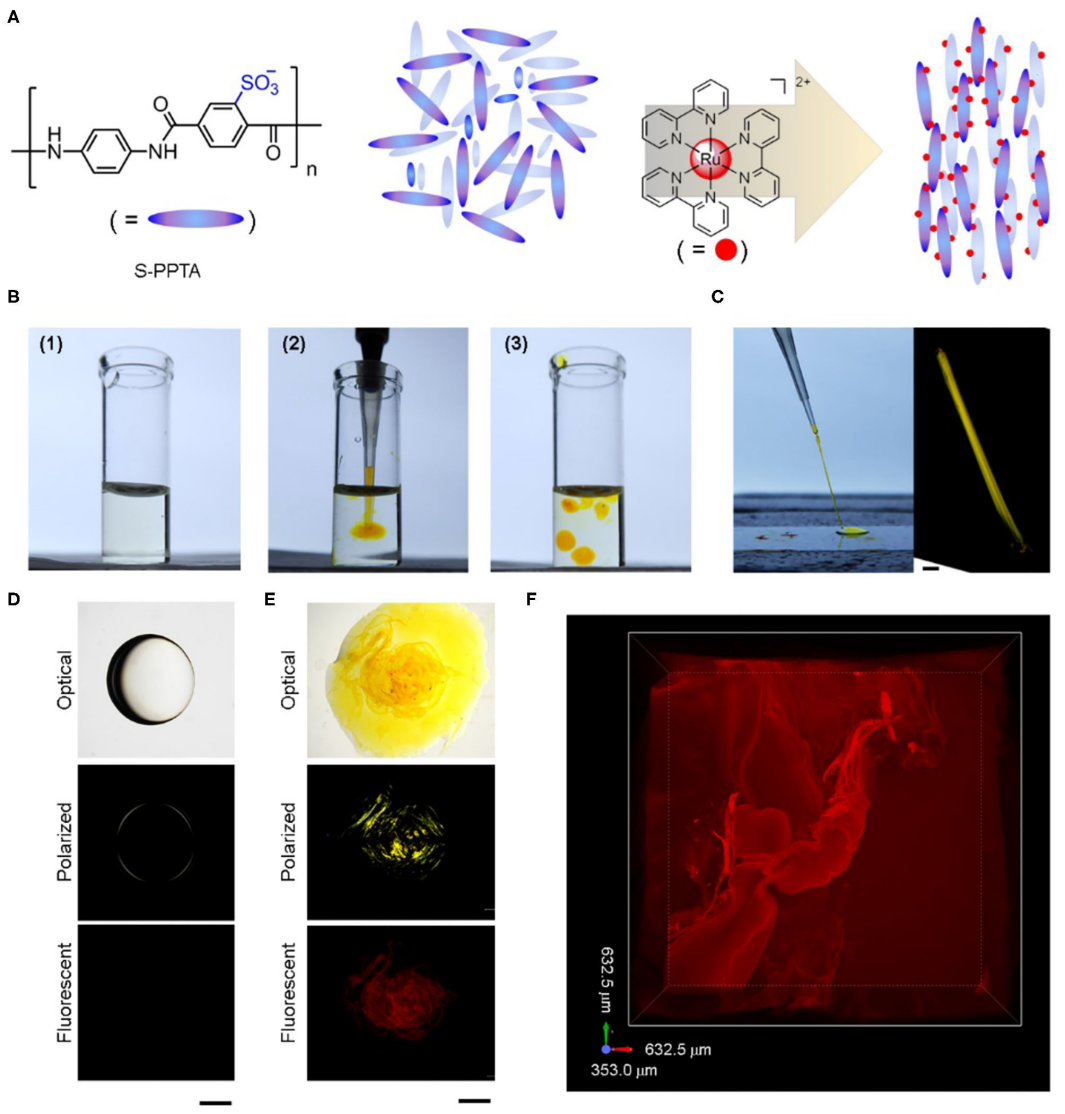
Construction of catalyst-embedded LLC gels. **(A)** Schematic illustration of lyotropic liquid crystal organized by electric charge attraction. The chemical structures of mesogen S-PPTA and catalyst ruthenium tris(bipyridine) are also shown. Counter chloride ions in catalyst are omitted for clarity. **(B)** Optical images of (1) S-PPTA solution in water, (2) formation of LLC gel through the injection of catalyst solution into S-PPTA solution, and (3) fabrication of individual LLC gel blocks. **(C)** Dragging of freshly made LLC block on glass cover slice forms aligned LLC string and the birefringence of the aligned string. Scale bar represents 100 μm. **(D,E)** Optical, polarized, and fluorescent images of S-PPTA solution droplet without catalyst **(D)** and LLC gel block containing catalyst **(E)**. Scale bars represent 1 mm. **(F)** Confocal fluorescent image of catalyst-embedded LLC gel. 3D maximum intensity projection view is shown.

Poly(*p*-phenylene-sulfoterephthalamide) (S-PPTA) is employed as LLC mesogen in this work (Figure 1A) due to (1) its high solubility in water, making it accessible and compatible to perform a BZ reaction in aqueous solution; (2) its lyotropic nematic behavior which is independent of the molar mass of the polymer (Viale et al., 2005); and (3) its negative surface charges, providing the electrical charge attraction for the positively charged metal catalyst, ruthenium(II) tris(bipyridine) chloride in this case. At low concentration, S-PPTA mesogens are in isotropic phase because the surface charges repulsively impede the alignment of mesogens. The introduction of catalyst not only helps overcome the charged repulsion of mesogen molecules, aligning mesogens for liquid crystal phase transition, but more importantly, the catalyst molecules non-covalently crosslink mesogens, driving the assembly of mesogens to form liquid crystalline gels. Compared with covalent immobilization of catalyst into a polymer chain, our approach of non-covalent catalyst embedment is time-saving and minimal in the preparation procedures.

### Preparation of Catalyst-Embedded LLC Gels

S-PPTA is dissolved in water to yield a slightly viscous solution at 0.25 wt.% concentration, which is lower than the reported critical gelation concentration (1.0 wt.%) (Ohsedo et al., 2015). LLC gel forms immediately by injecting catalyst solution into the above S-PPTA solution. The formed LLC gels stand isolated and can be manipulated individually inside the S-PPTA solution (Figure 1B). The LLC gels can be aligned and dragged to form strings (Figure 1C), showing promising potentials in programmable fabrication of different shapes. The dragged LLC strings as well as LLC gel blocks exhibit strong birefringence inside the gel under a polarized optical microscope (POM) (Figures 1C,E), whereas the S-PPTA solution droplet only shows weak birefringence on the edge (Figure 1D). Fluorescent images (Figures 1D,E) confirm the ruthenium(II)-based catalyst assists in forming LLC gels and reveal the microstructures inside LLC gels (Figure 1F).

**Figure 2:**
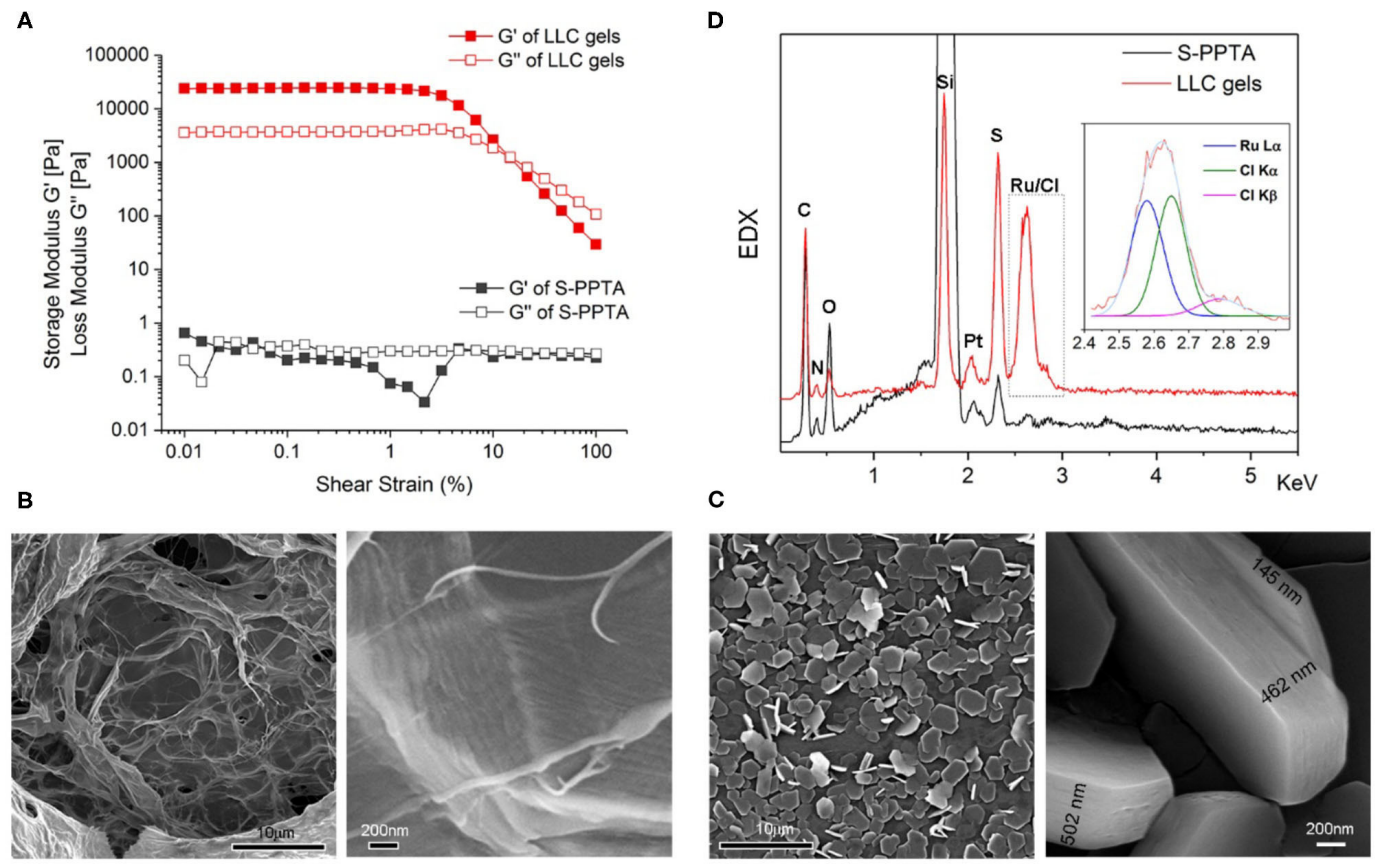
Characteristics of LLC gels. **(A)** Strain dependence of dynamic storage moduli (G′) and loss moduli (G″) of S-PPTA solution without catalyst (gray) or catalyst-embedded LLC gels (red). **(B,C)** SEM images of S-PPTA solution without catalyst **(B)** or catalyst-embedded LLC gels **(C)**. **(D)** Energy-dispersive X-ray spectroscopy (EDX) spectra of selected SEM images of S-PPTA (black) or catalyst-embedded LLC gels (red). The peaks at around 2.6 keV are split using multi-peaks fitting for Ru Lα and Cl Kα/Kβ.

The mechanical properties of catalyst-embedded LLC gels are evaluated. Rheological analysis indicates the S-PPTA solution is fluidic liquid at a concentration of 0.25 wt.%. In the presence of catalyst, the formed LLC gels show remarkable enhancement in the mechanical properties (Figure 2A). The storage modulus shows a higher than 4-order magnitude of enhancement; the loss modulus shows a higher than 3-order magnitude of enhancement.

Microstructures of catalyst-embedded LLC gels are also investigated via scanning electron microscopy (SEM). S-PPTA forms assembled films in solution. Zoomed-in SEM image indicates the films are organized by aligned filaments (Figure 2B). In the LLC gels, highly organized micro-disks with hexagonal shape are observed under SEM. High-resolution image from side view shows layer-by-layer assembly forming highly organized micro-disks with different thicknesses, ranging from 145 to 502 nm (Figure 2C). Energy-dispersive X-ray spectroscopy (EDX) spectra of selected SEM images (Figure 2D) show characteristic peaks of catalyst molecule in the assembled micro-disks of LLC gels. The energy of Ru Lα is observed at 2.578 keV (theoretically at 2.558 keV), and the energies of Cl Kα and Cl Kβ are observed at 2.648 and 2.782 keV (theoretically at 2.621 keV for Cl keV and at 2.812 keV for Cl Kβ, respectively). These results support our design of catalyst-embedded LLC gels through electric charge attraction.

### Chemical Oscillations in Catalyst-Embedded LLC Gels

Due to the easy processing properties of the catalyst-embedded LLC gels, we make LLC sticks by slowly dragging the freshly prepared LLC gel block using a pipette (Figure 1C and Supplementary Figure 1). The prepared LLC sticks are immersed in water overnight to remove unembedded catalyst. The LLC sticks with a diameter around 50–60 μm are cut into pieces with 1–2 mm length and immersed in BZ solution.

We first attempt to optimize BZ oscillations on the catalyst-embedded LLC gels. High concentration of NaBrO_3_ (0.5M) leads to the failure of BZ oscillation because of fast oxidation of catalyst; high concentration of malonic acid (0.3 M) generates many bubbles which seriously interfere with the reaction recording. Under low concentration BZ reaction ([NaBrO_3_] = 0.050 M, [malonic acid] = 0.021 M, [H_2_SO_4_] = 0.50 M), however, the catalyst is exchanged by protons and releases to the solution, resulting in fading and continuous shrinkage of liquid crystalline gels (Supplementary Figure 2).

**Figure 3:**
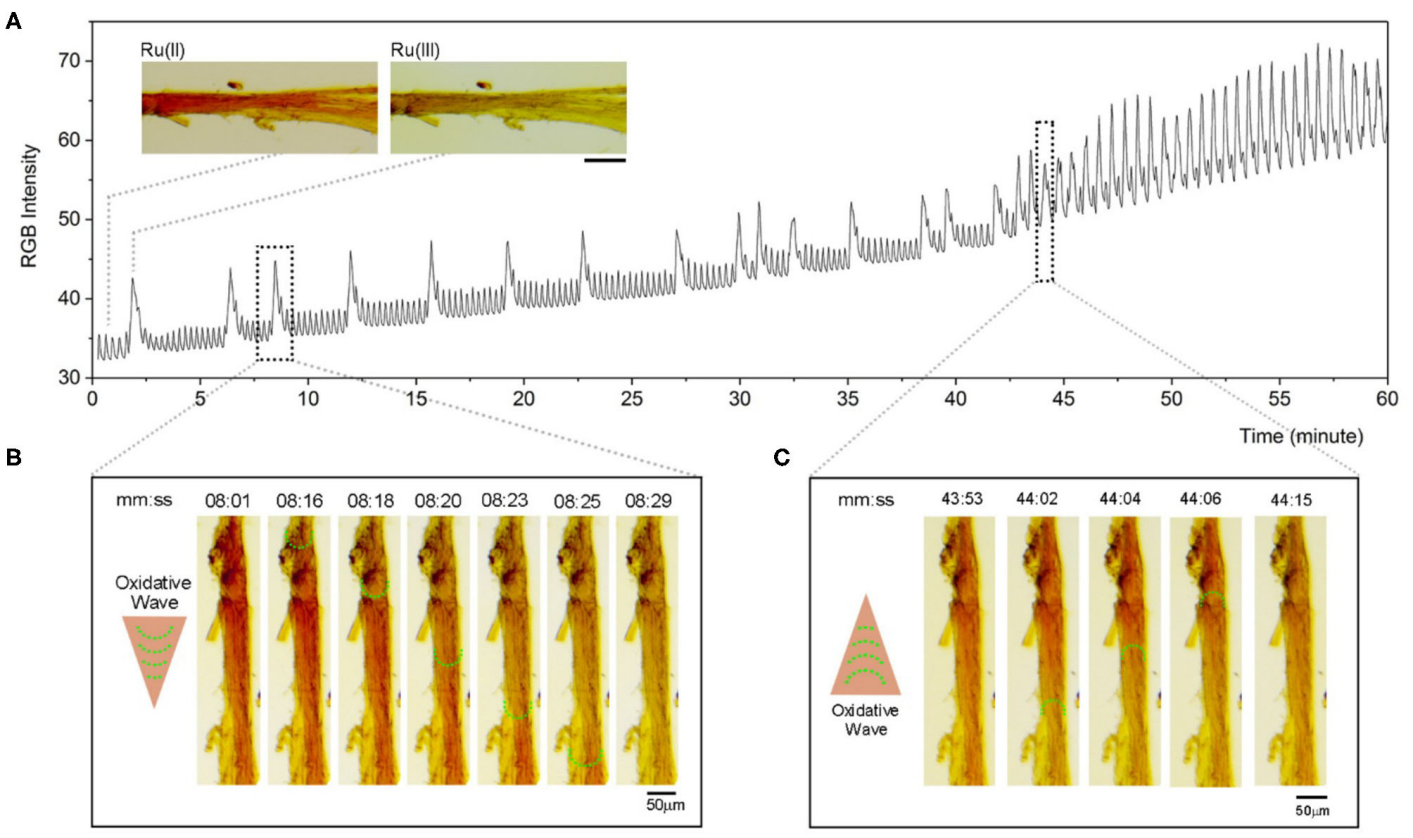
Chemical oscillations of catalyst-embedded LLC gels during BZ reaction. **(A)** Color intensity profile of LLC stick immersed in BZ solution ([H_2_SO_4_] = 0.5 M, [NaBrO_3_] = 0.28 M, [malonic acid] = 0.071 M). Inserted images show representative appearances of LLC stick in reduced state (Ru(II), red) or in oxidized state (Ru(III), light yellow). Scale bar represents 100 μm. **(B)** Selected optical images from the time period of 08:01–08:29 (mm:ss) show chemical waves along the LLC gel stick. Arrow and green dotted half-circles indicate the direction of oxidative waves along the stick. **(C)** Selected optical images from a time period of 43:53–44:15 (mm:ss) show chemical waves along the LLC gel stick. Arrow and green dotted half-circles indicate a reversed direction of oxidative waves along the stick. The images were rotated and cropped, and their brightness and contrast were adjusted by applying the same adjustment parameters to each image for clear illustration.

Optimized chemical condition ([NaBrO_3_] = 0.28 M, [malonic acid] = 0.071 M, [H_2_SO_4_] = 0.50 M) is applied to perform BZ reaction on the catalyst-embedded LLC gels. As shown in Figure 3, chemical oscillation occurs at 2 min, as observed in the changing color of the LLC stick switching between dark red [reduced state of the catalyst, Ru(II)] and light yellow [oxidized state of the catalyst, Ru(III)]. The color intensity profile is plotted (Figure 3A) and illustrates the co-existence of long-term chemical oscillations (2–5 min) contributed to the BZ patterns on LLC stick, and short-term chemical oscillations (10–15 s) contributed to the BZ patterns in the solution (Supplementary Figure 3). Within the first 42 min, the chemical waves are oscillating with a long period of 1–5 min; the oxidative waves spread continuously from the stick side that contains more catalyst, as indicated by the deeper red color. Representative series images from the period of 08:01–08:29 (Figure 3B) show that the direction of oxidative waves spreading is in accordance with the concentration gradient of Ru(II) catalyst. During the period of 43–60 min, however, the oscillation speeds up with a frequency of two oscillations per minute. Meanwhile, the reversed spreading direction of oxidative waves is observed (Figure 3C). These phenomena can be reasoned that the pool of Ru(II) catalyst stocked in the LLC stick has ran out.

In addition, the LLC stick shrinks instantly when the BZ solution is added, probably due to the exchange of catalyst by protons under strong acidic condition of BZ reaction. The observation of oscillating pattern in the solution supports the diffusion of catalyst out of the LLC stick. The size of LLC stick during the chemical oscillation is measured and the result shows a general decrease in the diameter of LLC stick (Supplementary Figure 4). Despite the general shrinkage of the LLC stick, we observe small-scale diameter oscillation (around 10%) during the long periodic chemical oscillation (0–42 min), which is synchronized with the chemical oscillation profile (Supplementary Figure 5). During the sped-up oscillation, no obvious oscillation in the diameter of LLC stick is observed. This finding that LLC stick swells during the oxidized Ru(III) state while it shrinks during the reduced Ru(II) state in a BZ reaction is in accordance with our previous reports (Zhang et al., 2012b, 2014) and other groups' reports (Yoshida and Ueki, 2014; Kim et al., 2017) on polymer gels covalently immobilized with catalyst.

Attempting to reason the acceleration of chemical waves and swelling of LLC sticks, we examine the SEM images of LLC gels after BZ reaction. Even though the mechanical properties of LLC gels after BZ reaction do not display significant differences compared with LLC gels before BZ reaction (Supplementary Figure 6), the micro-composites show a remarkably structural difference under SEM. Before the BZ reaction, the hexagonal micro-disks in LLC gels are highly organized and compact that are <4 μm in diameter (Figure 2C). After the BZ reaction, however, the micro-disks swell into individual unity with a diameter up to 7.5 μm, while the fibers are kept aligned in each assembled unity (Supplementary Figure 7). The diffusion rate of BZ reactive intermediates within loosely aligned fibers unities is much faster than within compactly packing plates, resulting in speeding up of the chemical wave propagation.

### Morphological Oscillation in Catalyst-Embedded LLC Gels

**Figure 4:**
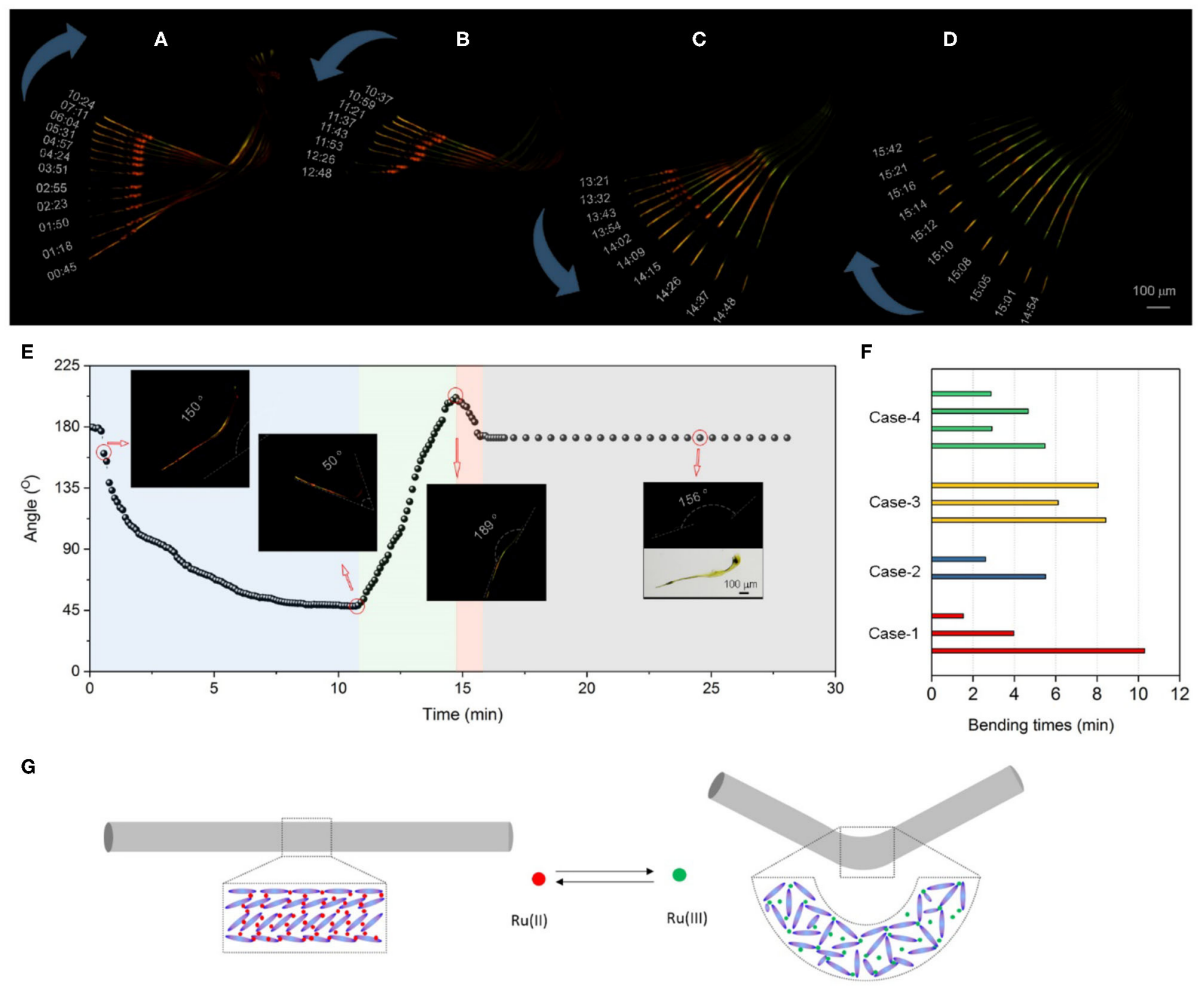
Mechanical oscillations of catalyst-embedded LLC gel during BZ reaction. **(A)** POM images at a certain time point show the morphological changes of LLC fiber immersed in BZ solution. **(A–D)** Superimposed POM images from the time period of **(A)** 00:45–10:24, **(B)** 10:37–12:48, **(C)** 13:21–14:48, and **(D)** 14:54–15:42 (mm:ss) of LLC fiber in BZ solution. The blue arrows indicate the morphological oscillation of bending **(A)**, unbending **(B)**, straightening **(C)**, and re-bending **(D)** in BZ solution. **(E)** The plot of fiber angles versus time. Inserted images show representative POM images of fiber at a certain time point. **(F)** Histogram of fiber bending duration time in four cases**. (G)** Schematic drawing of assembly transition from liquid crystal phase to isotropic phase that bends a LLC fiber.

SEM images of LLC sticks verify at the microscopic level our hypothesis of BZ reaction driving the mechanical response of LLC gels. The macroscopic swelling–shrinkage of LLC stick, however, is covered up by the continuous shrinkage of LLC gels during BZ reaction. To amplify the chemomechanical oscillations, we make catalyst-embedded LLC fibers with a smaller diameter (around 30 μm) by stretching the freshly prepared catalyst-embedded LLC strings. SEM images show the highly aligned fibers on the stretched LLC fibers (Supplementary Figure 8). POM images of LLC fibers (Figure 4) show stronger birefringence than LLC string (Figure 1C), indicating a more tightly assembled liquid crystal phase in the stretched LLC fibers.

When the small LLC fiber is immersed into BZ solution, it rapidly responds to the chemical waves and begins to bend at as early as 45 s. The bending rate slows down and stops at 10:24 (Figure 4A). The internal bending angle changes from 135 to 50°. The unbending process is observed from the time period of 10:37–12:48 accompanied with the fading birefringence (Figure 4B), suggesting a transition from compact liquid crystal phase to a loose packing phase in the LLC fiber. The internal bending angle expands to 72°. In the period of 13:21–14:48, the unbending process continues. LLC fiber straightens to 189° with an increasing birefringence in the LLC fiber (Figure 4C). Then a rapid bending process occurs again and completes within 1 min in the period of 14:54–15:42 (Figure 4D). At the final stage, the birefringent intensity decreases, and the LLC fiber turns green, suggesting the catalyst has been completely oxidized into Ru(III) and the BZ reaction stops. The plot of angles versus time (Figure 4E) clearly shows the LLC fiber bends three times within 16 min driving by a BZ reaction, and stops bending after 16 min. Similar results are observed (Supplementary Figure 9), suggesting that macroscopic mechanical oscillator of LLC gels is successfully realized in response to the chemical waves in BZ reaction. The bending duration time of LLC fibers with an average bending cycle of 1.5 (bend–unbend–bend) ranges from 1.6 to 10 min (Figure 4F).

The design of liquid crystalline network as stimuli-responsive actuators takes full advantages of the phase transition of molecular assembly (White and Broer, 2015; Kumar et al., 2016). We propose that the mechanical oscillations in LLC gels are due to the transition of molecular assembly between liquid crystal phase and isotropic phase (Figure 4G). Initial catalyst-embedded LLC gels before BZ reaction show bright birefringence under a POM. Also, SEM images show crystalline mesogen packing, suggesting the liquid crystal phase assembly of reduced Ru(II) catalyst with S-PPTA mesogen. During BZ reaction, catalyst is oxidized to form Ru(III). The charge change alters local balance and reorganizes the assembly of surrounding mesogens, which turns into isotropic phase. POM images show that the birefringence of LLC fibers vanishes at the bending site, confirming the isotropic phase transition during mechanical oscillations.

## Conclusion

Liquid crystalline gels integrating actuating molecules responsive to external fields (light, heat, magnetic fields, electric fields) have achieved great successes in stimuli-responsive materials (White and Broer, 2015; Kumar et al., 2016; Gelebart et al., 2017). As proof of concept, we demonstrate a simple and convenient method of constructing catalyst-embedded LLC gels and achieve self-oscillating LLC gels by converting chemical waves in a BZ reaction. Our approach of non-covalent crosslinking embedment of catalyst into LLC gels minimizes the preparation procedures, allowing quick conceptual tests with the lowest cost of time. The catalyst-embedded LLC gels are easily processable, capable of different shapes of fabrication. We have successfully achieved both chemical oscillation and mechanical oscillation on LLC gels. Our results suggest the applications of self-oscillating liquid crystalline gels driven by BZ reaction, and also increase the diversity of autonomous soft matter. More efforts are being put into this work to gain insight into more molecular clues on how the mesogen orientation and liquid crystal phase transition change as a consequence of oxidation–reduction oscillation of metal catalyst in BZ reaction. It should be noted that the stability of this non-covalent embedment of catalyst shows limitations in deep development of BZ-reaction-driving autonomous liquid crystal actuators. An improved version of continuous self-oscillating LLC gels is under development.

## Methods

### Materials and Instruments

^1^H NMR and ^13^C NMR spectra are acquired on an AVANCE 400 (400 MHz; Bruker) spectrometer. The ^1^H NMR and ^13^C NMR chemical shifts (δ) are given in parts per million referring to internal standard tetramethylsilane (TMS). Rheological measurements of the moduli under varying strain are performed using an MCR 302 rheometer (Anton-Paar) at 25 °C with a cone plate (24.977 mm diameter) at a gap of 0.105 mm and ω of 10 1/s. SEM images are captured using Quanta250 FEG scanning electron microscope (FEI) at 10 kV; EDX spectra are recorded at 20 kV. The SEM samples are placed on silicon wafer and coated with a thin layer of Pt after freeze-drying. The freshly prepared LLC gel block is placed on a glass cover-slide and imaged by a confocal laser scanning microscope (A1; Nikon). Filters are set as ex/em 488/600 ± 20 nm. BZ reaction on the LLC gels is performed at 25 °C under an SMZ18 stereomicroscope (Nikon). Time-lapse optical images or POM images are captured every 2 s to record the chemical oscillations.

### Synthesis of S-PPTA

S-PPTA is synthesized according to literature method (Viale et al., 2005). ^1^H NMR (400 MHz, DMSO-d6) δ 10.96 (1H, s), 10.55 (s, 1H), 8.72 (d, J = 4.84 Hz, 1H), 8.41 (s, 1H), 8.04 (s, 1H), 7.89–7.55 (m, 4H). ^13^C NMR (101 MHz, DMSO-d6) δ 157.47, 156.97, 144.95, 144.24, 143.34, 130.98, 129.92, 128.98, 126.87, 126.58, 121.44, 120.71, 119.50, 115.66.

### Preparation of LLC Gels

Twenty-five milligrams of S-PPTA is dissolved in 10 ml water at 70°C, then allowed cool to r.t. to yield a 0.25 wt.% solution. A stock solution of 5 mM Ru(II) tris(bipyridine) chloride is prepared in water. To a solution of 500 μl S-PPTA in a glass vial, 20 μl of catalyst stock solution is slowly injected. Orange LLC gel block forms immediately. To make LLC sticks, a freshly prepared LLC gel block is dragged slowly out of S-PPTA solution using a pipette to yield a LLC string, which is allowed to hang in the air for a few minutes until the dragged string is stiff enough to keep its shape when moving the pipette. The LLC sticks are immersed in water overnight to remove unembedded catalyst. To make small LLC fibers, a freshly dragged LLC string is stretched using a pipette on a glass slice. The stretched strings are allowed dry in the air to yield small stiff fibers, which are immersed in water overnight to remove unembedded catalyst.

### Performance of BZ Reaction on LLC Gels

The LLC sticks or LLC fibers are cut into pieces with 1–2 mm length and placed on a glass-bottom dish under a stereo microscope. BZ solution is used as [H_2_SO_4_] = 0.5 M, [NaBrO_3_] = 0.050 M, [malonic acid] = 0.021 M for low concentration condition; [H_2_SO_4_] = 0.5 M, [NaBrO_3_] = 0.50 M, [malonic acid] = 0.10 M or [H_2_SO_4_] = 0.5 M, [NaBrO_3_] = 0.20 M, [malonic acid] = 0.30 M for high concentration condition; [H_2_SO_4_] = 0.5 M, [NaBrO_3_] = 0.28 M, [malonic acid] = 0.071 M for optimized condition, respectively. Optical images or POM images are captured every 2 s. To analyze chemical oscillations, RGB color intensities of selected ROIs on the LLC stick vs. time are recorded and plotted. For morphological analysis, time-lapsed images are loaded into ImageJ and the “Analyze Particles” analytic package is used to quantify the area of LLC sticks which is divided by length to yield the average diameter of LLC sticks. The calculated average diameters vs. time are plotted. The angles of bending fibers are measured in ImageJ.

## Data Availability Statement

All datasets generated for this study are included in the article/Supplementary Material.

## Author Contributions

GL and YZ contributed to conception, design of the study, and wrote the article. GL, WC, and QZ performed the experiments and analyzed data. All authors contributed to article revision, and read and approved the submitted version.

## Conflict of Interest

The authors declare that the research was conducted in the absence of any commercial or financial relationships that could be construed as a potential conflict of interest.
