# Supplementary material for: Chemical Oscillation and Morphological Oscillation in Catalyst-Embedded Lyotropic Liquid Crystalline Gels
Source: Front Chem. 2020 Oct 23;8:583165. doi: 10.3389/fchem.2020.583165 (PMC7645047; doi:10.3389/fchem.2020.583165)
Supplement: Supplementary file 1 [file Table_1.DOCX]

Supplementary Material

Chemical Oscillation and Morphological Oscillation in Catalyst-embedded Lyotropic Liquid Crystalline Gels

Guanying Li^1^, William Cortes^1^, Qizheng Zhang^1^, Ye Zhang^1^*

^1^ Bioinspired Soft Matter Unit, Okinawa Institute of Science and Technology Graduate University, Okinawa, Japan

*** Correspondence:**Ye Zhang
ye.zhang@oist.jp


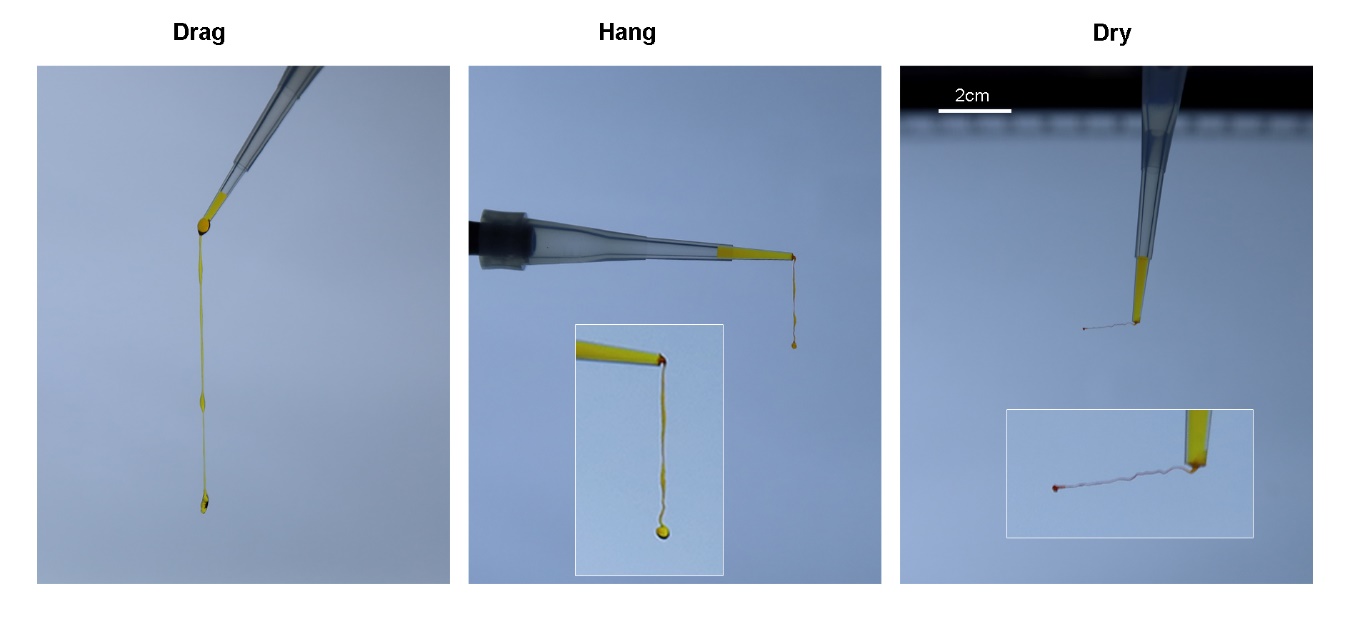


**Supplementary Figure 1.** Fabrication of LLC sticks. A freshly prepared LLC gel block is dragged slowly using a pipette to form a hanging string. The dragged LLC sting is allowed to hang in the air for a few minutes until the string is stiff enough to keep its shape when moving the pipette.


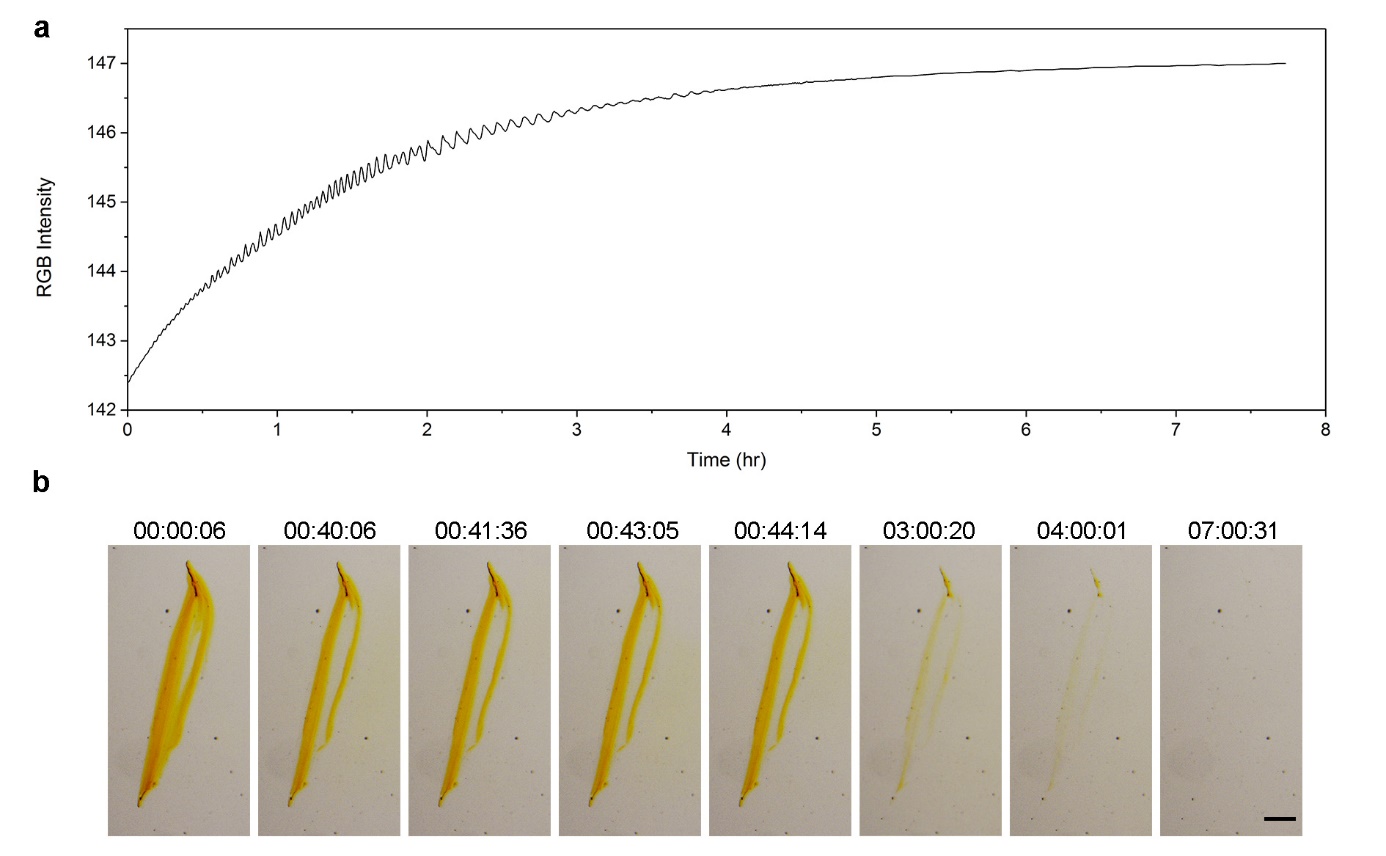


**Supplementary Figure 2. (a)** RGB intensity profile of LLC gel immersed in BZ solution([H_2_SO_4_] = 0.5M, [NaBrO_3_] = 0.05M, [malonic acid] = 0.021M). (b) Representative optical images at certain reaction time-point. Image at 00:00:06 (hh:mm:ss) shows initial image of catalyst-embedded LLC gels immersed in BZ solution. Images from 00:40:06 to 00:44:14 (hh:mm:ss) show a cycle of BZ oscillation indicated by the intensity profile (**a**). Image at 03:00:20 (hh:mm:ss) shows the fading of LLC gel color, suggesting the release of catalyst. Image at 04:00:01 (hh:mm:ss) shows the continuous fading of LLC gel color. Image at 07:00:31 (hh:mm:ss) shows the colorless fiber after BZ reaction, suggesting the complete release of catalyst.


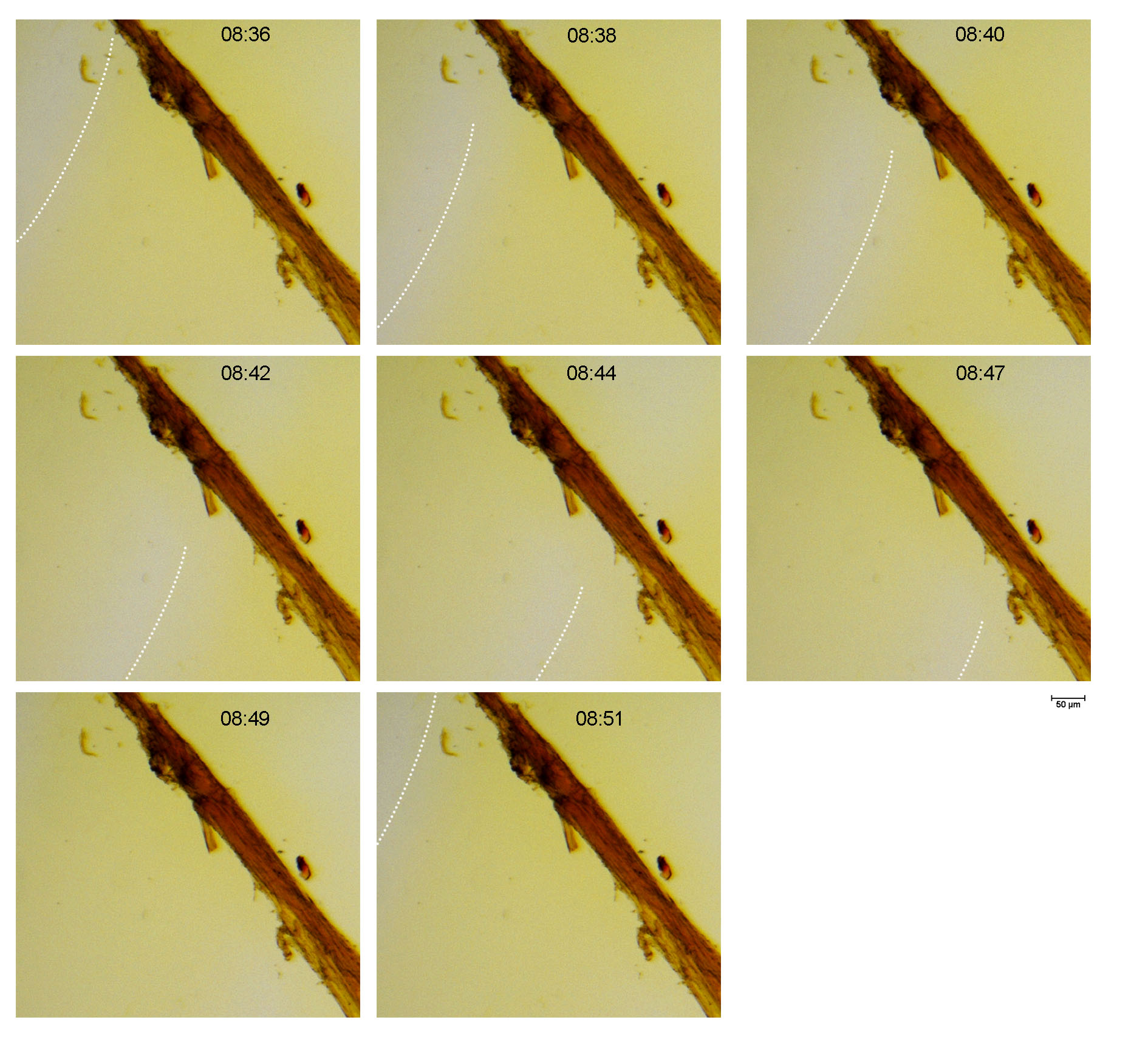


**Supplementary Figure 3.** Short duration chemical oscillations referred to **Figure 3a**. Selected optical images from a time period of 08:36 - 08:51 (mm:ss) show the fast chemical waves in the solution, meanwhile no color changes occurred on the LLC stick. The brightness and contrast were adjusted to show the color of released catalyst in the solution. Dotted curves indicated the oxidative patterns in the solution. Scale bar represents 50 μm.


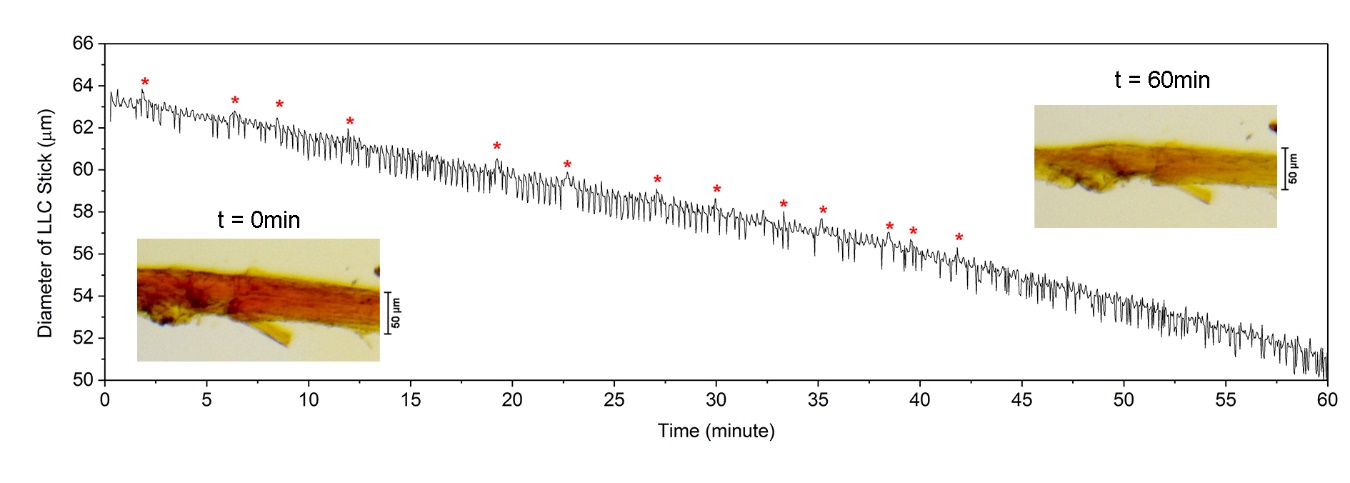


**Supplementary Figure 4.** Profile of the average diameter of LLC stick during a BZ reaction. Red stars indicate the swelling periods. Inserted images show LLC stick at t = 0 min and t = 60 min.


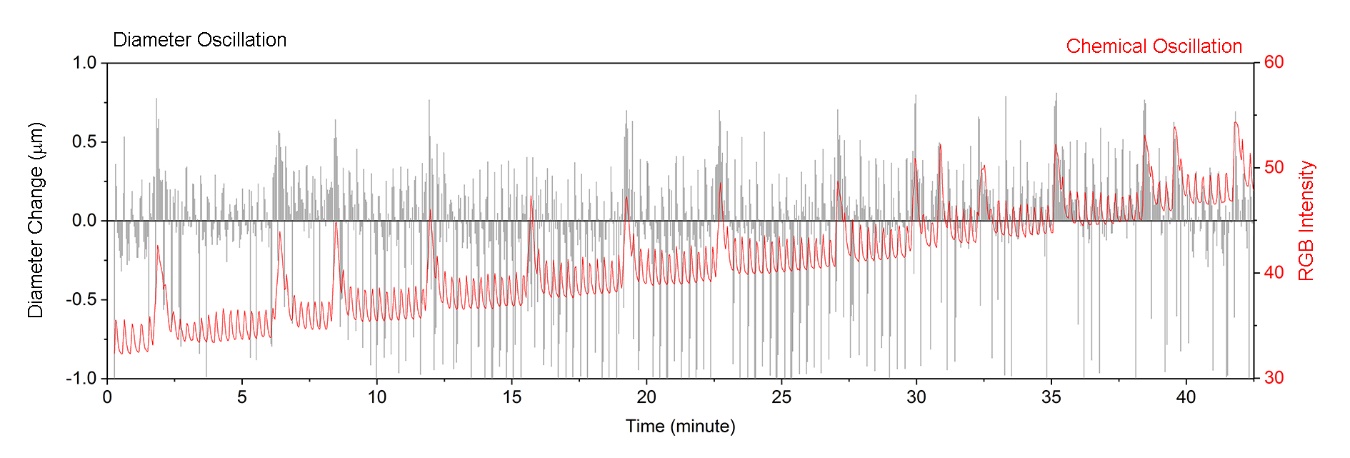


**Supplementary Figure 5.** Overlaid profiles of the diameter oscillation (grey) of LLC stick and chemical oscillation (red) during a BZ reaction on the LLC stick.


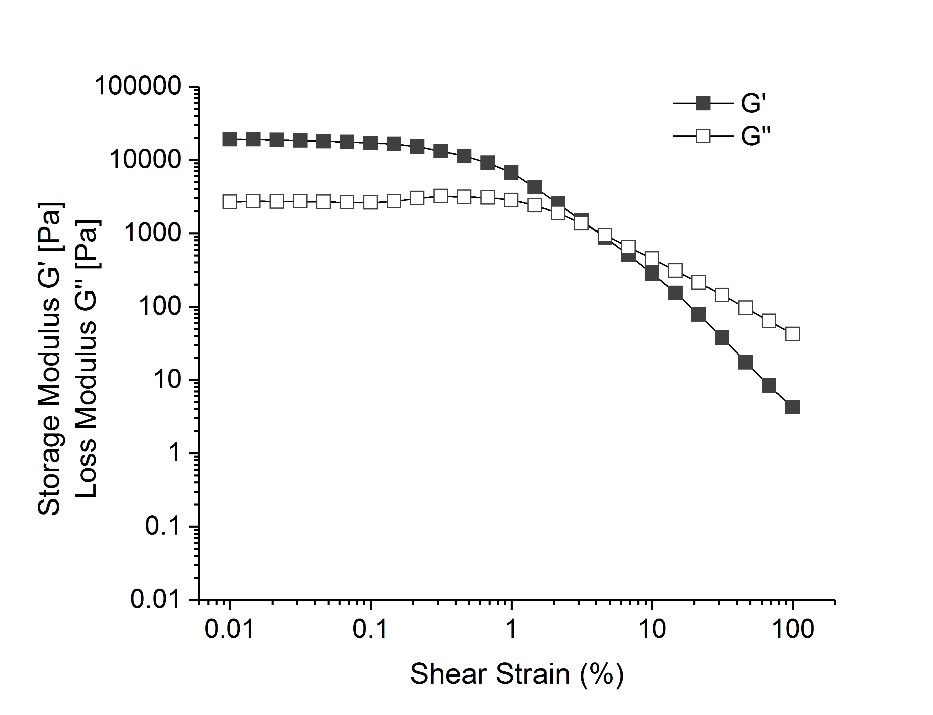


**Supplementary Figure 6.** Rheological analysis of LLC gels after BZ reaction.


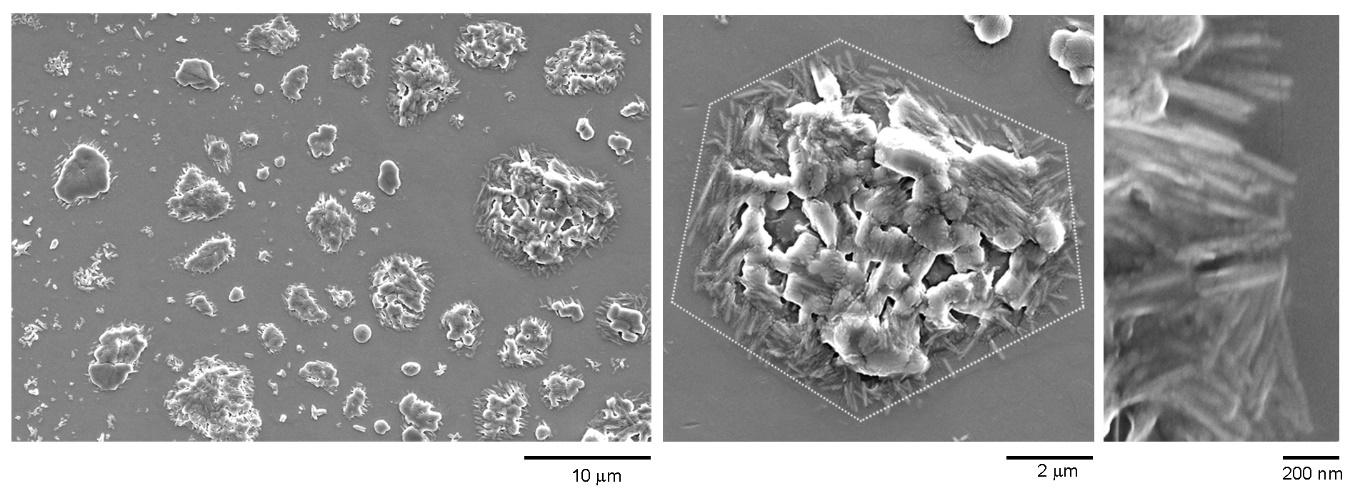


**Supplementary Figure 7.** SEM images of LLC gels after BZ reaction.


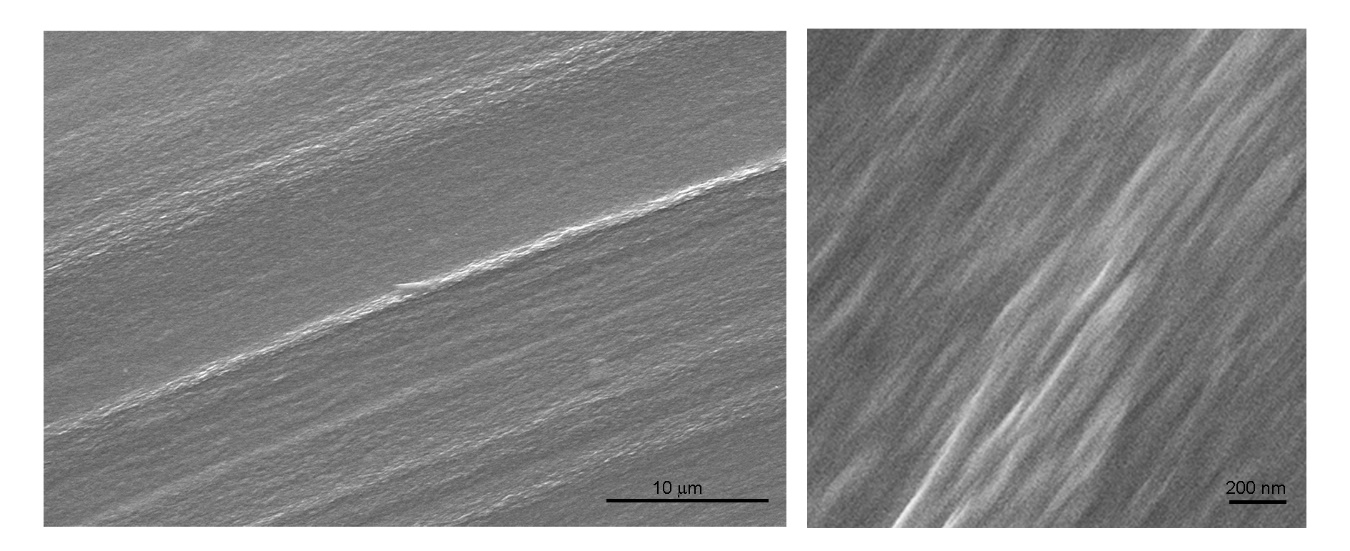


**Supplementary Figure 8.** SEM images stretched LLC fibers.


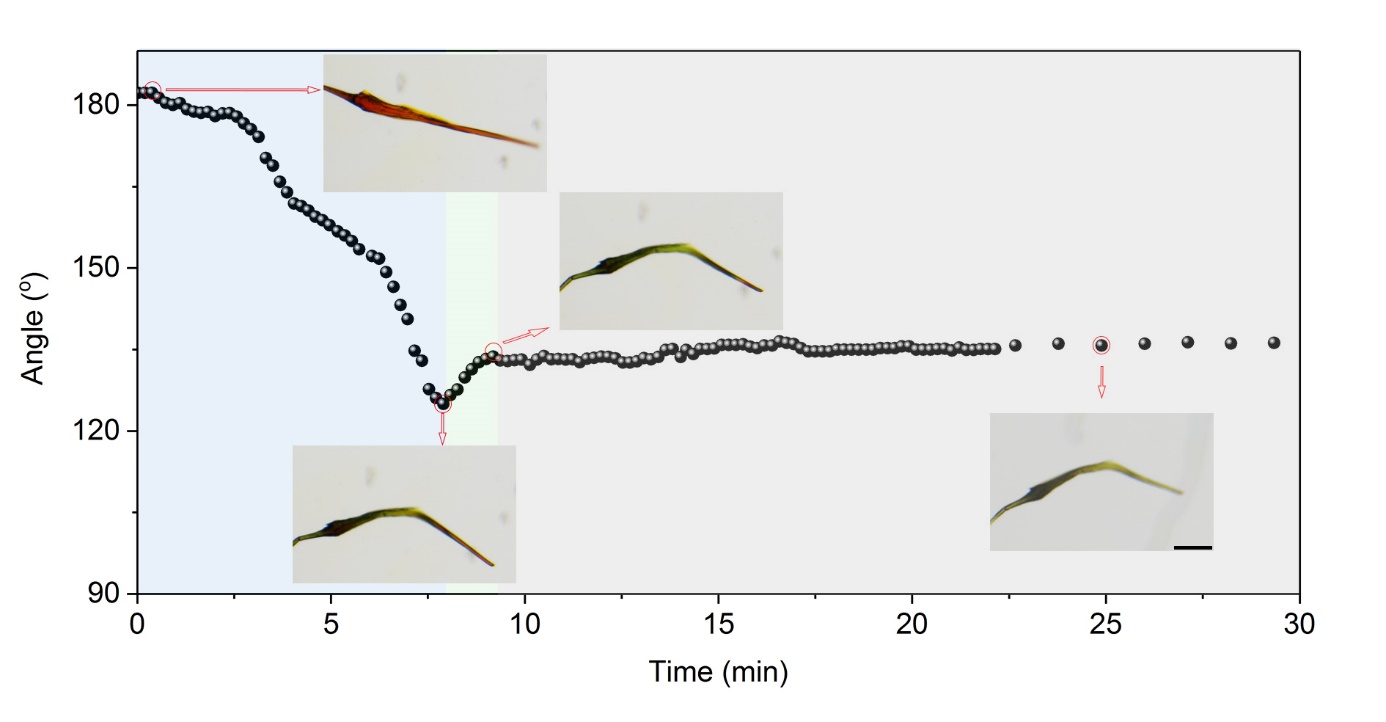


**Supplementary Figure 9.** The angles vs time plot of a bending fiber in a repeated experiment. Inserted images show representative optical images of fiber at certain time-point. Scale bar represents 100 μm.
